# The Effects of Temperature and Anesthetic Agents on Ciliary Function in Murine Respiratory Epithelia

**DOI:** 10.3389/fped.2014.00111

**Published:** 2014-10-16

**Authors:** Adam B. Christopher, Sebastian Ochoa, Evonne Krushansky, Richard Francis, Xin Tian, Maliha Zahid, Ricardo Muñoz, Cecilia W. Lo

**Affiliations:** ^1^Department of Developmental Biology, School of Medicine, University of Pittsburgh, Pittsburgh, PA, USA; ^2^Department of Pediatrics, School of Medicine, University of Pittsburgh, Pittsburgh, PA, USA; ^3^Office of Biostatistics Research, National Heart Lung and Blood Institute, Bethesda, MD, USA

**Keywords:** ciliary beat frequency, dexmedetomidine, fentanyl, isoflurane, hypothermia

## Abstract

**Background:** Mucus transport mediated by motile cilia in the airway is an important defense mechanism for prevention of respiratory infections. As cilia motility can be depressed by hypothermia or exposure to anesthetics, in this study, we investigated the individual and combined effects of dexmedetomidine (dex), fentanyl (fen), and/or isoflurane (iso) at physiologic and low temperatures on cilia motility in mouse tracheal airway epithelia. These anesthetic combinations and low temperature conditions are often used in the setting of cardiopulmonary bypass surgery, surgical repair of congenital heart disease, and cardiac intensive care.

**Methods:** C57BL/6J mouse tracheal epithelia were excised and cilia dynamics were captured by videomicroscopy following incubation at 15, 22–24, and 37°C with different combinations of therapeutic concentrations of dex (10 nM), fen (10 nM), and iso (0.01%). Airway ciliary motion was assessed and compared across conditions by measuring ciliary beat frequency and ciliary flow velocity. Statistical analysis was carried out using unpaired *t*-tests, analysis of variance, and multivariate linear regression.

**Results:** There was a linear correlation between cilia motility and temperature. Fen exerted cilia stimulatory effects, while dex and iso each had ciliodepressive effects. When added together, fen + iso, dex + iso, and dex + fen + iso were all cilia inhibitory. In contrast fenl + dex did not significantly alter ciliary function.

**Conclusion:** We show that ciliary motility is stimulated by fen, but depressed by dex or iso. However, when used in combination, ciliary motility showed changes indicative of complex drug–drug and drug–temperature interactions not predicted by simple summation of their individual effects. Similar studies are needed to examine the human airway epithelia and its response to anesthetics.

## Introduction

Multi-ciliated epithelial cells in the respiratory tract help maintain pulmonary health by clearing mucus and expelling particulates that have the potential to cause infection and inflammation (1). This process is mediated by motile cilia in the respiratory epithelia that beat in synchrony to propel mucus from the airway. Forward flow required to achieve effective mucociliary clearance is influenced by many factors that can affect ciliary motility. Previous studies have shown that ciliary beat frequency (CBF) is tightly correlated to temperature, exhibiting a sigmoidal pattern with limited effects above physiologic temperature and below ~5–10°C (2–5). Multiple pharmaceutical agents have also been investigated for their effects on ciliary motion. Of particular interest are inhaled and intravenous anesthetics. Volatile anesthetics, such as isoflurane (iso), have been shown to depress CBF and inhibit mucociliary clearance (6–12). One study demonstrated that commonly used intravenous anesthetics, such as ketamine and fentanyl (fen) used at high doses, can have a ciliostimulatory effect, whereas others such as midazolam, propofol, and dexmedetomidine (dex) had no effect on CBF (13).

In clinical scenarios, where intravenous and inhaled anesthetics are used in combination with hypothermia, such as in patients with congenital heart disease (CHD) undergoing cardiopulmonary bypass surgery (CBS), insights on the combined effects of low temperature and different anesthetic combinations on airway ciliary function are of critical importance for optimizing surgical and postsurgical care. Of particular interest are CHD patients, where recent evidence has shown that patients with CHD associated with heterotaxy have a high incidence of ciliary dysfunction similar to those associated with primary ciliary dyskinesia (PCD) (14, 15). Such CHD patients may be at increased risk for pulmonary complications post-operatively due to anesthetic-mediated mucociliary clearance function associated with respiratory ciliary dysfunction (16).

In this study, we investigated the effects of temperature and various combinations of anesthetics on ciliary motility using mouse tracheal epithelia. Our studies focused on iso, fen, and dex anesthetics commonly used in our institution during cardiopulmonary bypass. Dex, a commonly used anesthetic during the operative and post-operative period, is an α_2_-adrenergic receptor agonist with analgesic, sedative, and anxiolytic properties (17). Dex is extensively used in recent years due to its ability to produce effective sedation with few adverse effects, but little is known about how it affects ciliary function and respiratory outcome (18–20). We also examined the effects of fen, an opioid receptor agonist commonly used for sedation and analgesia during cardiac surgery. We quantitatively assessed CBF and flow velocity with exposure to different drug combinations. We found drug–drug and drug–temperature interactions on ciliary function that provide an insight on the complex regulation of ciliary motility by anesthetics and temperature. Our findings provide a better understanding of anesthetic-mediated impairment of ciliary function during hypothermic conditions, and could serve as basis for human studies, which could lead to better selection of the anesthetic/analgesic combinations to optimize mucociliary clearance function and possibly respiratory outcome in high risk patients.

## Materials and Methods

### Mouse trachea sample preparation and video microscopy

Experiments were conducted in accordance with an animal protocol approved by the Institutional Animal Care and Use Committee of the University of Pittsburgh. After euthanasia, mouse trachea were removed, flushed extensively, and prepared according to our previously published protocol (21, 22). Each sample was placed luminal side down on a glass slide in L-15 medium containing 0.50-μm Fluoresbrite microspheres (Polysciences, Inc., Warrington, PA, USA). Cilia dynamics were captured with a 100× differential interference contrast (DIC) oil objective and a Leica DMIRE2 inverted microscope (Leica Microsystems). Movies [400 frames/s (fps)] were recorded with a Phantom v4.2 camera (Vision Research, Wayne, NJ, USA). For analysis at 15°C, the entire experiment was performed in a cold room set at 15°C, while 37°C incubation was carried out using a heated stage and an objective heater (Bioptechs, Butler, PA, USA).

### Anesthetic concentration

Treatment media containing anesthetics was prepared by adding the appropriate dose of each drug to L-15 medium + 10% fetal bovine serum. Dex and fen were used at concentrations based on titration studies and published therapeutic plasma concentrations of 1–8 nM for dex (23) and 3–100 nM for fen (24). Iso was added to a concentration of 0.01% by volume (0.8 mM) using previously established partition coefficient (PC) of iso (25) in Tyrode’s solution in 0.1% BSA at 37°C. This most closely simulated the liquid bathing, the ciliated airway epithelia. Assuming the delivery of 2× the minimal alveolar concentration of iso (MAC = 1.2% atm), we can substitute the PC into the following equation and determine an aqueous concentration to be 0.88mM, or 0.01% by volume of stock solution: *C*_aq_ (mM) = 1000/22.4 * *p*(% atm)/100 * PC (26).

### Quantitative measurement of ciliary beat frequency and cilia-generated flow

To measure CBF and cilia-generated flow, at least two videos were collected from each tracheal sample, with a total of three different tracheas sampled at each condition. A line was traced perpendicular to the cilia along that each video was resliced to create a kymograph using ImageJ (NIH). The CBF was then measured for an average of eight “areas of interest” (AOI) from different tracheas by calculating the mean of four successive wavelengths per cilia using the program GIMP v2.6. Therefore, the average CBF for 48 AOI was calculated for most treatment conditions (except 37°C, for which only the first video of each sample was analyzed, yielding an average of 24 cilia). To calculate cilia-generated flow speed, each of the two videos per trachea was examined at a reduced frame rate of 15 fps. Five beads at equal distance from each tissue sample were then tracked using DIAS v3.4.2 (Soll Technologies, Inc., Iowa City, IA, USA) through 20 consecutive frames to yield an average speed and directionality for 30 beads per condition (15 beads at 37°C conditions).

### Real time PCR analysis

Mouse tracheal epithelia were allowed to reciliate in culture using a suspension culture method that enriched for respiratory epithelial cells RNA was extracted from tracheal epithelia and cDNA amplified (NuGen Ovation Kit) (27). Real time PCR was performed on a Perkin Elmer 7500HT thermal cycler using primers specific for mouse α- and β-adrenergic receptors as well as opioid receptors. All expression levels were normalized using β-actin.

### Statistical analysis

Ciliary beat frequency (Hz) and flow speed (μm/min) are expressed as mean ± standard deviation. Values of *n* represent the numbers of cilia or beads analyzed for CBF and flow speed, respectively, for each treatment condition. Log-transformed flow speed was used in the statistical tests to reduce the variability and improve normality of the data. Difference in the mean values between two conditions was determined by a heteroscedastic unpaired *t*-test. The one-way analysis of variance models was used to examine differences in the eight anesthetic groups at each temperature and differences in the three temperatures given the same anesthetic treatment. Then at each temperature, a multivariate linear regression model was used to assess the main effect of the three anesthetics and their interactions. Finally, an overall multivariate regression model that included data from three temperatures was used to estimate the main temperature and anesthetic effects and identify significant drug–drug and drug–temperature interactions. The non-significant interaction terms were dropped in the regression models by the backward model selection procedure. All tests were two-tailed and *P* values <0.05 were considered significant. Analyses were performed with SAS 9.3 (SAS Institute, Cary, NC, USA).

## Results

**Figure 1:**
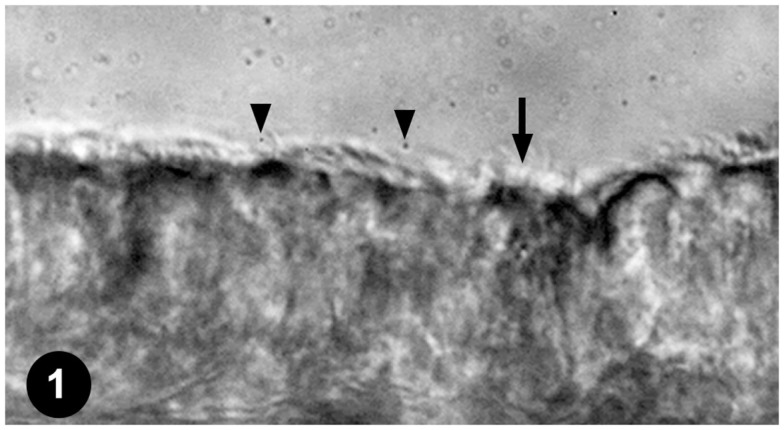
**Mouse tracheal ciliated respiratory epithelia**. Ciliated tracheal epithelium (arrow) bathed in medium containing microspheres (arrowheads) for quantifying cilia-generated flow.

To assess the effects of anesthetics on respiratory airway cilia function, strips of mouse trachea were imaged by videomicroscopy to capture cilia dynamics (Figure 1). The videos obtained were quantitatively assessed for ciliary motility by measuring CBF, and cilia-generated flow was quantified by adding fluorescent beads to the media and measuring fluorescent bead displacement velocity. CBF and cilia-generated flow speed were used to assess the effects of temperature and anesthetic exposure on cilia motility.

### Effect of temperature on airway ciliary motility

**Figure 2:**
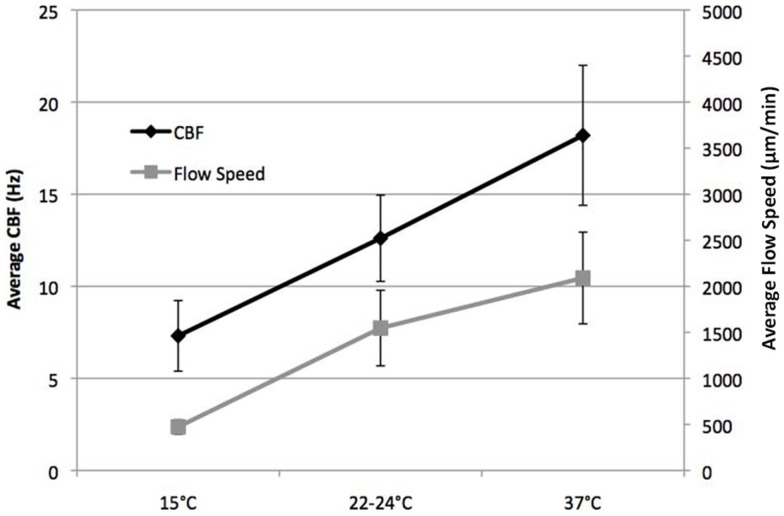
**Effect of temperature on ciliary beat frequency (CBF) and flow velocity**. CBF and flow velocity at 15, 22–24, and 37°C.

We examined temperature effects on ciliary motility at 37°C (physiological body temperature), 22–24°C (room temperature), and 15°C (cardiopulmonary bypass temperature). As temperature declined from 37 to 25°C, CBF and flow speed decreased (Figure 2), dropping from 18.2 ± 3.8 at 37°C to 12.6 ± 2.3 Hz at room temperature (22–24°C; *P* < 0.001). This was associated with a change in flow speed from 2090 ± 498 to 1547 ± 410 μm/min (*P* = 0.001), respectively (Figure 2). Further reduction in temperature to 15°C depressed CBF to 7.3 ± 1.9 Hz (*P* < 0.001) and flow speed to 473 ± 78 μm/min (*P* < 0.001). Overall, we observed a linear decrease in temperature and flow speed with reduction in temperature.

### Titration of anesthetic effects on airway cilia motility

**Figure 3:**
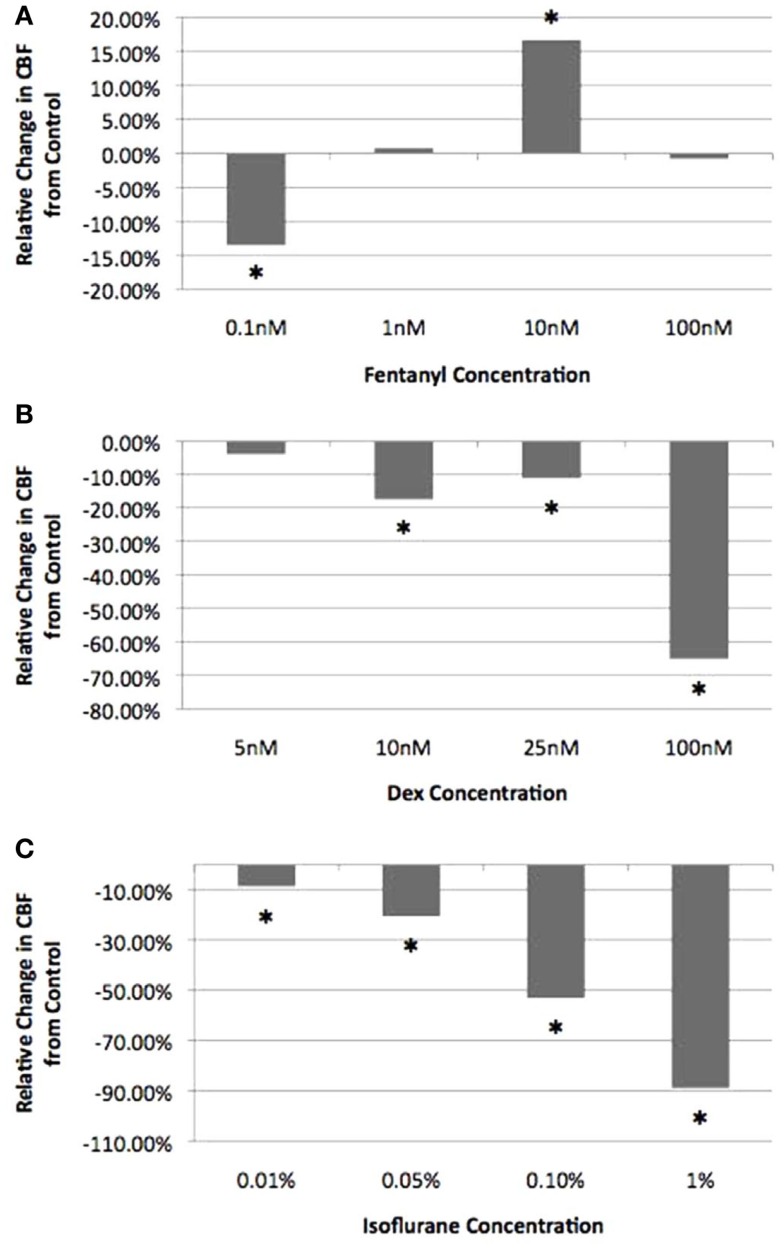
**Titration of anesthetic concentration on ciliary beat frequency**. Cilia beat frequency was measured at room temperature (22–24°C) with increasing concentrations of fen **(A)**, dex **(B)**, and iso **(C)**. Values are expressed as percentage difference from control at 22–24°C (12.6 ± 2.3 Hz). The lowest drug concentration exerting a significant effect on CBF and was within the clinical dose was chosen for all further experimental analyses (10 nM fen, 10 nM dex, 0.01% iso). *indicates significant difference from control (*P* < 0.05).

To assess the effects of fen, dex, and iso on airway cilia motility, we first carried out a titration analysis with different anesthetic concentrations spanning the established clinical doses (Table S1 in Supplemental Material). For the inhaled anesthetic iso, the clinically relevant dose was calculated based on the expected solubility of the inhaled iso gas in airway mucus (see “Materials and Methods”). Titration analysis of fen at room temperature (22–24°C) showed a cilia inhibitory effect at very low concentration of 0.1 nM (CBF = 12.6 ± 2.3 Hz). In contrast, at 10 nM, which is equivalent to a 1 μg/kg dose, a significant stimulatory effect was observed with CBF of 14.7 ± 3.1 Hz (*P* < 0.001) (Figure 3). In comparison, dex and iso each depressed CBF at all concentrations, with the first significant effect noted at 10 nM dex, equivalent to a dose of 1 μg/kg (10.4 ± 2.4 Hz, *P* < 0.001) and at 0.01% iso, or 2× MAC (11.5 ± 2.2 Hz, *P* = 0.011) (Figure 3). For the remainder of the studies below, we used iso (0.01%; 2× MAC), dex (10 nM), and fen (10 nM) at the clinically established doses to examine their effects on cilia motility in isolation and in combination with each other at the three different temperatures.

### Opposing effects of fentanyl vs. dexmedetomidine and isoflurane on ciliary motility

**Figure 4:**
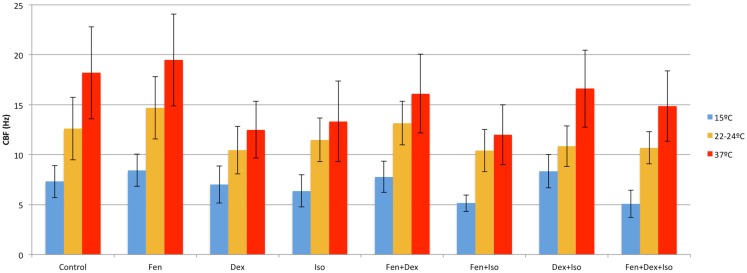
**Anesthetic effects on ciliary beat frequency**. Mean CBF after exposure to different combinations of anesthetics at 15, 22–24, and 37°C.

**Figure 5:**
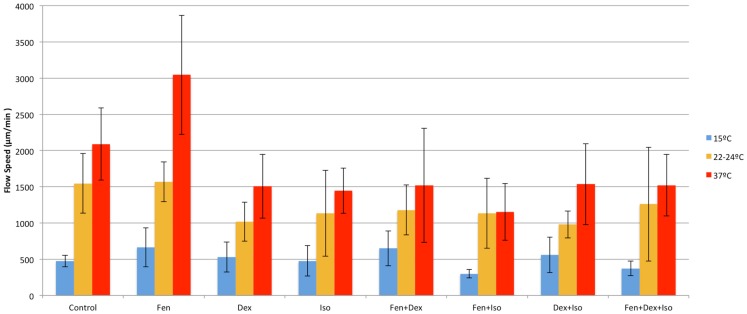
**Anesthetic effects on cilia flow velocity**. Mean cilia flow velocity after exposure to different combinations of anesthetics at 15, 22–24, and 37°C.

**Figure 6:**
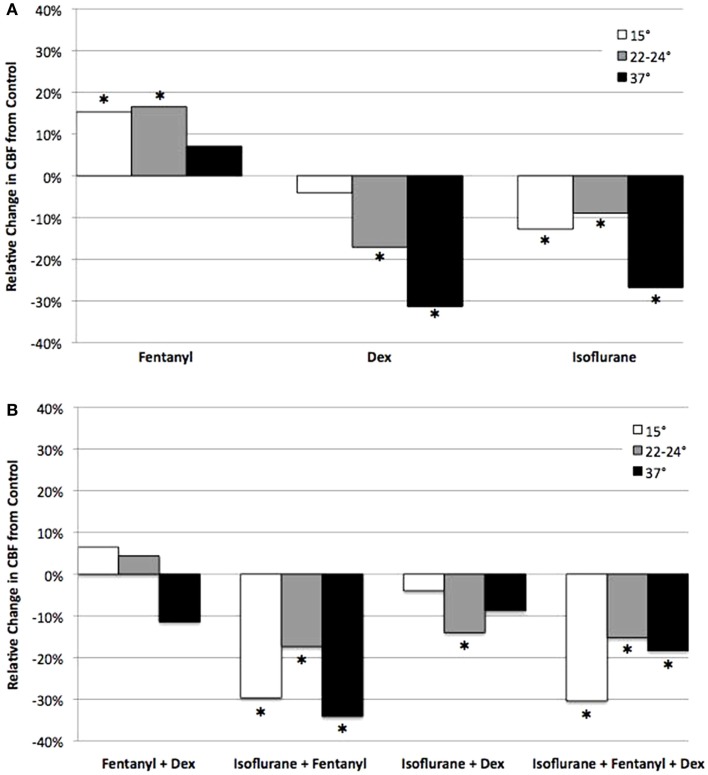
**Analysis of anesthetic effects on ciliary beat frequency relative to control**. The effects of anesthetics alone **(A)**, and in different combinations **(B)** on CBF at 15, 22–24, and 37°C relative to control. *indicates significant difference from control (*P* < 0.05).

**Figure 7:**
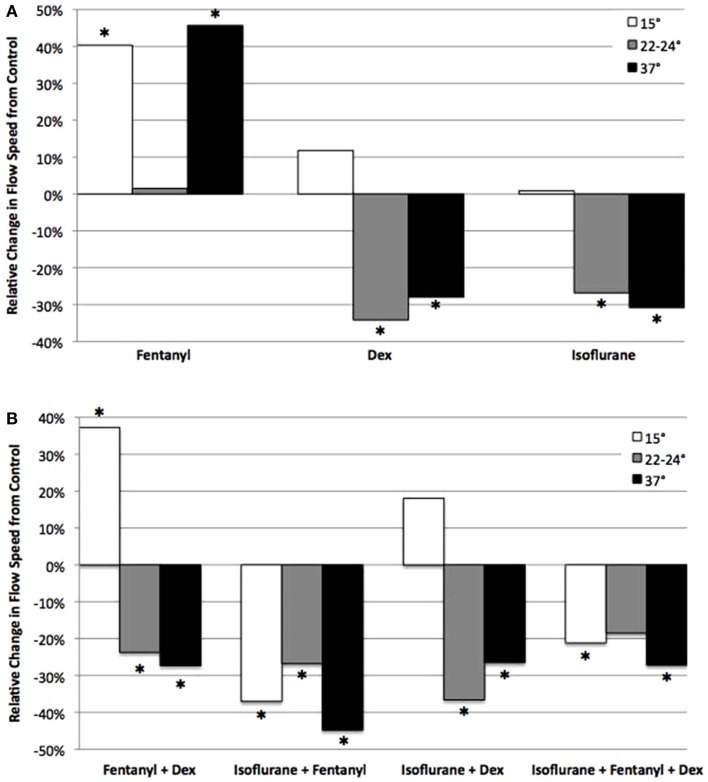
**Analysis of anesthetic effects on cilia-generated flow relative to control**. The effects of anesthetics alone **(A)**, and in different combinations **(B)** on flow speed at 15, 22–24, and 37°C relative to control. *indicates significant difference from control (*P* < 0.05).

Fen at 10 nM increased CBF and flow speed at all three temperatures (Figures 4 and 5). Of note, with hypothermia (15°C), fen raised the mean CBF by 15% from 7.3 ± 1.9 to 8.4 ± 1.6 Hz (*p* = 0.003) (Figure 6), and with an even greater effect on flow speed with a 40% increase from 473 ± 78 to 637 ± 271 μm/min (*p* = 0.003) (Figure 7). In contrast, dex or iso administered at 10 nM or 0.01% respectively, exerted largely inhibitory effects on CBF and flow, with the greatest inhibitory effects noted at 22–24 and 37°C (Figures 4–7).

### Combined effects of anesthetics and temperature on ciliary motility

The opposing effects of fen vs. dex or iso would suggest that ciliary function might be restored if dex or iso were used in combination with fen. This was in fact observed with therapeutic dosing of fen and dex together, which preserved CBF at levels similar to that of untreated control at all three temperatures (Figure 6). This was associated with an increase in flow speed at 15°C (37% increase, *p* < 0.001), but flow speed was decreased at 22–24 and 37°C (27% decrease at 37°C, *p* = 0.013) (Figure 7). In contrast, exposure to iso-fen, or iso-dex, or all three together, uniformly depressed both CBF and flow (Figures 6 and 7). However, the magnitude of cilia inhibition was quite different from that expected by simple summation of their effects. Fen, which enhances ciliary motility, when used in combination with iso depressed ciliary function to levels lower than that with iso alone. This was significant for CBF and flow speed across all temperatures (Figures 6 and 7). In comparison, dex and iso, which individually were cilia inhibitory, together caused depression of CBF to levels similar to or less than that observed with either anesthetic alone (Figures 6 and 7), suggesting the suppressive effects of the two drugs were reduced when they were used in combination. These findings showed dex and fen exerted unique interactions with iso.

### Analysis of drug–drug and drug–temperature interactions on cilia motility

To examine possible anesthetic–anesthetic and anesthetic–temperature interactions, we used the totality of over 700 CBF/flow measurements generated above for linear regression modeling (Tables 1 and 2). From the analysis of CBF and flow speed, we observed dex–fen together exerted only additive effects without any additional drug–drug interactions (Tables 1 and 2). In contrast, fen–iso and dex–iso combinations showed opposing drug–drug interactions (Tables 1 and 2). Thus, the cilia-stimulatory effect exerted by fen was depressed by significant negative interaction with iso, while positive interaction between iso and dex attenuated their individual cilia-inhibitory effects (Tables 1 and 2).

**Table 1:** **Parameter estimate in ANOVA models for ciliary beat frequency**.

Variables	Model A		Model B		Model C		Model D	
	Temperature 15°C		Temperature 22–24°C		Temperature 37°C		Overall model	
	(*n* = 417)		(*n* = 399)		(*n* = 207)		(*n* = 1023)	
	Estimate (SE)	*P*-value	Estimate (SE)	*P*-value	Estimate (SE)	*P*-value	Estimate (SE)	*P*-value
Intercept (control)	7.40 (0.22)	<0.0001	12.46 (0.27)	<0.0001	17.58 (0.66)	<0.0001	17.83 (0.40)	<0.0001
Fen	0.93 (0.24)	0.0001	2.40 (0.32)	<0.0001	2.53 (0.74)	0.008	2.03 (0.38)	<0.0001
Dex	−0.46 (0.24)	0.06	−1.84 (0.32)	<0.0001	−4.46 (0.74)	<0.0001	−4.42 (0.49)	<0.0001
Iso	−0.84 (0.30)	0.006	−1.19 (0.39)	0.002	−4.17(0.89)	<0.0001	−4.64 (0.51)	<0.0001
**Drug–drug interaction**
Fen and iso	−2.55 (0.34)	<0.0001	−3.02 (0.46)	<0.0001	−4.05 (1.05)	0.0002	−3.04 (0.31)	<0.0001
Dex and iso	0.80 (0.34)	0.02	1.64 (0.46)	0.0003	7.55 (1.05)	<0.0001	7.49 (0.69)	<0.0001
**Temperature**
15°C	–	–	–	–	–	–	−10.55 (0.49)	<0.0001
22–24°C	–	–	–	–	–	–	−5.37 (0.48)	<0.0001
**Drug and Temperature Interactions (reference category: 37°C)**								
15°C/fen	–	–	–	–	–	–	−0.85 (0.42)	0.045
15°C/dex							3.99 (0.60)	<0.0001
22–24°C/dex	–	–	–	–	–	–	2.58 (0.60)	<0.0001
15°C/iso	–	–	–	–	–	–	4.04 (0.61)	<0.0001
22–24°C/iso	–	–	–	–	–	–	3.46 (0.60)	<0.0001
15°C/dex and iso							−6.74 (0.84)	<0.0001
22–24°	–	–	–	–	–	–	−5.84 (0.85)	<0.0001
/dex and iso								

*Note: At each temperature, a full regression model including three two-way interactions and three-way interactions among fentanyl (fen), dexmedetomidine (dex), and isoflurane (iso) were performed first, and then the non-significant terms were dropped in the final regression model presented in the table. The overall model used all data and included the main temperature and drug effects and all the significant interactions, with temperature 37°C as the reference category*.

**Table 2:** **Parameter estimate in ANOVA models for flow speed (log-transformed)**.

	Model A		Model B		Model C		Model D	
	15°C		22–24°C		37°C		Overall model	
	(*n* = 240)		(*n* = 250)		(*n* = 130)		(*n* = 620)	
	Estimate (SE)	*P*-value	Estimate (SE)	*P*-value	Estimate (SE)	*P*-value	Estimate (SE)	*P*-value
Intercept (control)	6.12 (0.05)	<0.0001	7.28 (0.05)	<0.0001	7.61 (0.08)	<0.0001	7.73 (0.07)	<0.0001
Fentanyl	0.22 (0.06)	0.0004	0.09 (0.05)	0.05	0.37 (0.12)	0.002	0.15	0.0002
							−0.04	
Dexmedetomidine	0.12 (0.04)	0.008	−0.37 (0.06)	<0.0001	−0.32 (0.12)	0.008	−0.57 (0.09)	<0.0001
Isoflurane	−0.02 (0.06)	0.71	−0.40 (0.06)	<0.0001	−0.36 (0.11)	0.002	−0.51 (0.10)	<0.0001
**Drug–drug interaction**
Iso and fen	−0.59 (0.09)	<0.0001	–	–	−0.62	0.0002	−0.30 (0.09)	0.002
					−0.16	
Iso and dex	–	–	0.37 (0.09)	<0.0001	0.39 (0.16)	0.02	0.75 (0.12)	<0.0001
Fen and dex	–	–	–	–	−0.47	0.0046	–	–
					−0.16	
Fen and dex and iso	–	–	–	–	0.69 (0.23)	0.003	–	–
**Temperature**
15°C	–	–	–	–	–	–	−1.54 (0.08)	<0.0001
22–24°C	–	–	–	–	–	–	−0.47 (0.08)	<0.0001
**Drug and temperature Interactions (reference category: 37°C)**								
15°C and dex	–	–	–	–	–	–	0.62 (0.11)	<0.0001
22–24°C and dex	–	–	–	–	–	–	0.20 (0.11)	0.054
15°C and iso	–	–	–	–	–	–	0.39 (0.12)	0.001
22–24°C and iso	–	–	–	–	–	–	0.14 (0.12)	0.23
15°C and fen/iso	–	–	–	–	–	–	−0.22 (0.11)	0.043
22–24°C and	–	–	–	–	–	–	0.24 (0.11)	0.023
Fen/ISO	
15°C and dex/iso	–	–	–	–	–	–	−0.61 (0.15)	<0.0001
22–24°C and dex/iso	–	–	–	–	–	–	−0.37 (0.15)	0.013

*Note: Log-transformed flow speed was used in the models. At each temperature, a full regression model including three two-way interactions and three-way interactions among fentanyl (fen), dexmedetomidine (dex), and isoflurane (iso) were performed first, and then the non-significant terms were dropped in the final regression model presented in the table. The overall model used all data and included the main temperature and drug effects and all the significant interactions, with temperature 37°C as the reference category*.

The linear regression analysis was carried out using 37°C as the reference temperature to model how lower body temperature may affect the effects of drugs on ciliary motility, revealing significant drug–temperature interactions. Dex or iso each showed positive temperature interaction with both 15 and 22–24°C, causing a greater depression of CBF and flow speed when compared to their individual effects at 37°C (Table 2). Fen exhibited negative interaction with 15°C, but this was only observed for CBF and not for flow speed. When combined, dex–iso showed negative temperature interaction at both 15 and 22–24°C, which was observed for both CBF and flow speed (Table 1). In contrast, fen–iso exhibited negative temperature interaction at 15°C but positive interaction at 22°C, which was only observed for flow speed but not CBF. When all three anesthetics were combined, no drug or temperature interactions were observed.

### Adrenergic and opioid receptor transcript expression in the mouse airway epithelia

**Figure 8:**
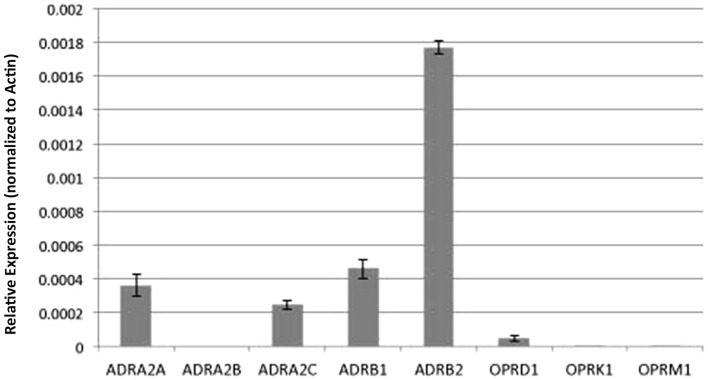
**Analysis of adrenergic and opioid receptor transcript expression in the mouse airway epithelia**. Real time PCR analysis of the mouse airway epithelia of various receptors (normalized to actin expression) showed expression of adrenergic but not opiate receptor transcripts.

To gain further insights into the possible molecular basis for the differing drug–drug and drug–temperature interactions on cilia function, we used real time PCR analysis to examine transcript expression for the adrenergic and opioid receptor subtypes in the mouse airway epithelia. Our analysis revealed robust expression of the β-adrenergic type 2 receptor and lower expression of the β type 1 receptors, but no transcripts were detected for the α-adrenergic or any of the opioid receptors (Figure 8).

## Discussion

We examined mouse trachea ciliary motility at different temperatures and with varying combinations of dex, fen, and iso to model conditions experienced during hypothermia associated with cardiac bypass and administration of multiple anesthetics. We found CBF generally correlated with flow speed, although the magnitude of the change can differ. This likely reflects the fact that CBF does not assess the ciliary waveform nor the coordination of the ciliary beat, which together determine flow. Consistent with previous studies, CBF and flow declined as temperature decreased from 37 to 22–24/15°C (2–5). While fen was cilia stimulatory, iso and dex were cilia inhibitory across all temperatures.

When these three anesthetics were used in different combinations, except for fen + dex, ciliary function showed non-additive drug–drug and drug–temperature interactions not predicted by simple summation of their individual effects. Fen + dex had additive effects, with fen counteracting the cilia inhibitory effects of dex. However, fen + iso exhibited negative interaction with more cilia inhibition, while iso + dex showed positive interaction with less cilia inhibition. Interestingly, when all three anesthetics were added together, no drug interactions were observed. Drug–temperature interactions were also observed for some of the drug combinations, with dex and iso each exhibiting positive temperature interaction with less cilia inhibition at 15 and 22–24°C, while fen showed modest negative temperature interaction at 15°C. In contrast, dex + iso showed strong negative temperature interaction with increased cilia inhibition at 15 and 22–24°C. Interestingly, when all three drugs were combined, no drug–temperature interactions were observed.

In preliminary studies to investigate the mechanism of anesthetic action, we examined transcript expression for the adrenergic receptors using real time PCR analysis. While we detected transcripts for β-adrenergic receptors, no transcripts were detected for either the α2 or μ opioid receptors known to mediate the effects of dex (28) and fen (24), respectively. As the RNA used in our analysis was obtained from reciliated airway epithelia largely devoid of other cell types in the trachea, this discrepancy suggests the primary responder to the anesthetic exposure may not be the ciliated epithelial cells in the trachea. Consistent with this possibility, studies by Iida et al. (13) using cultured rat respiratory epithelial cells showed dex at 10 and 100 nM had no effects on cilia motility and only slight stimulation with fen at 100 nM. The contrasts with the robust effects, we observed with dex and fen using intact mouse trachea.

Overall, our study suggests mucociliary clearance function can be better managed and optimized with careful consideration of the drug–drug and drug–temperature interactions on respiratory ciliary motility. The applicability of these findings in the mouse respiratory epithelia to ciliary function in the human airway needs to be carefully examined and validated. Future efforts to develop therapeutic strategies for optimizing mucociliary clearance function in patients undergoing surgical intervention will also require insights into the underlying pharmacology and identification of the primary responding cells in the airway.

## Conflict of Interest Statement

The authors declare that the research was conducted in the absence of any commercial or financial relationships that could be construed as a potential conflict of interest.

## Supplementary Material

The Supplementary Material for this article can be found online at http://www.frontiersin.org/Journal/10.3389/fped.2014.00111/abstract
