# Supplementary material for: The Effects of Temperature and Anesthetic Agents on Ciliary Function in Murine Respiratory Epithelia
Source: Front Pediatr. 2014 Oct 16;2:111. doi: 10.3389/fped.2014.00111 (PMC4199259; doi:10.3389/fped.2014.00111)
Supplement: Supplementary file 1 [file Data_Sheet_1.PDF]

Supplemental Table 1. Dose response titration for individual anesthetics at 22-24°C

Anesthetic Treatment	Average CBF (Hz)	Standard Deviation
Control	12.6	2.3
Fen 0.1nM	10.9	3.7
Fen 1nM	12.7	4.3
Fen 10nM	14.7	3.1
Fen 100nM	12.5	2.8
Dex 5nM	12.1	1.9
Dex 10nM	10.4	2.4
Dex 25nM	11.2	1.7
Dex 100nM	4.4	0.3
Iso 0.01%	11.5	2.2
Iso 0.05%	10.0	2.1
Iso 0.1%	5.9	1.6
Iso 1%	1.4	0.3

Supplemental Statistical Methods

Tukey’s multiple comparisons for eight drug groups at each temperature*

CBF : Temp=15

Tukey Grouping	Mean	N	Drug
A	8.4279	47	2.Fe
A			
B A	7.7828	46	5.FP
B			
B C	7.3081	45	1.CT
B C			
B C	7.0206	77	6.PI
B C			
B C	7.0123	65	3.Pr
C			
C	6.3741	47	4.Is
D	5.1440	44	7.FI
D			
D	5.0884	46	8.FPI

CBF : Temp=22-24

Tukey Grouping	Mean	N	Drug
A	14.6944	49	2.Fe
B	13.1585	56	5.FP
B			
C B	12.6076	53	1.CT
C			
C D	11.4784	53	4.Is
D			
D	10.8393	46	6.PI
D			
D	10.6866	48	8.FPI
D			
D	10.4497	47	3.Pr
D			
D	10.4207	47	7.FI

CBF :Temp=37

Tukey Grouping	Mean	N	Drug
A	19.487	24	2.Fe
A			
B A	18.195	24	1.CT
B A			
B A C	16.608	24	6.PI
B C			
B D C	16.111	32	5.FP
D C			
E D C	14.861	23	8.FPI
E D			
E D	13.324	32	4.Is
E			
E	12.496	24	3.Pr
E			
E	11.994	24	7.FI

Log(Flow): Temp=15

Tukey Grouping	Mean	N	Drug
A	6.41905	30	5.FP
A			
B A	6.37984	30	2.Fe
B A			
B A C	6.23219	30	6.PI
B A C			
B A C	6.20554	30	3.Pr
B C			
B D C	6.14673	30	1.CT
D C			
D C	6.07492	30	4.Is
D			
E D	5.88754	30	8.FPI
E			
E	5.67973	30	7.FI

Log(Flow): Temp=22-24

Tukey Grouping	Mean	N	Drug
A	7.34271	30	2.Fe
A			
B A	7.30313	40	1.CT
B			
B C	7.03082	30	5.FP
C			
C	6.99326	30	8.FPI
C			
C	6.95355	30	7.FI
C			
C	6.89798	30	4.Is
C			
C	6.89181	30	3.Pr
C			
C	6.87034	30	6.PI

Log(Flow): Temp=37

Tukey Grouping	Mean	N	Drug
A	7.9824	15	2.Fe
B	7.6142	15	1.CT
B			
C B	7.3276	15	6.PI
C B			
C B	7.2955	15	3.Pr
C B			
C B	7.2908	15	8.FPI
C			
C	7.2551	20	4.Is
C			
C	7.1929	20	5.FP
C			
C	6.9994	15	7.FI

*Means with the same letter are not significantly different.
